# Neural bases of consciousness in Zen meditation: central executive and default mode networks during cognitive conflict

**DOI:** 10.3389/fpsyg.2026.1774227

**Published:** 2026-04-10

**Authors:** Naoyuki Osaka, Takehiro Minamoto, Ken Yaoi, Miyuki Azuma, Mariko Osaka

**Affiliations:** 1 Department of Psychology, Kyoto University, Kyoto, Japan; 2 Department of Psychology, Faculty of Human Sciences, Shimane University, Matsue, Shimane, Japan; 3 Department of Psychology, Teikyo University, Otsuka, Hachioji, Japan; 4 Center for Information and Neural Networks, National Institute of Information and Communications Technology, Osaka, Japan

**Keywords:** attention control, central executive network, consciousness, default mode network, self recognition, Stroop test, Zen meditation

## Abstract

Consciousness in Zen meditation has garnered considerable interest in psychology and neuroscience, particularly in relation to its association with enhanced self-awareness. Zen meditation fosters social harmony by addressing mental and social conflicts, linking individuals to various societal aspects through the practice. This meditation, rooted in Buddhism, aims to perceive thoughts without judgment by attentively focusing on the present. However, the neural mechanisms behind its benefits have remained elusive. To investigate, we used functional magnetic resonance imaging on two groups: Zen monks with extensive meditation experience (average 7.53 years) and a control group without meditation background. Both groups completed the Stroop cognitive conflict task followed by meditation. Zen meditation, when performed by skilled monks, improved responses and cognitive control, and reduced cognitive conflict in the lateral and medial prefrontal cortices compared to the control group. Conflict tasks are generally associated with increased activity in the central executive network (CEN), whereas activity in the medial prefrontal cortex, a core component of the default mode network (DMN), has been linked to internally oriented processes such as self-referential thought and mind-wandering. The involvement of the DMN during meditation, however, appears to depend on the specific meditation style and cognitive state, with prior studies reporting mixed findings. These networks are often considered to exhibit an antagonistic relationship, with task engagement typically accompanied by CEN activation and concurrent DMN suppression. However, Stroop conflict trials in the present study did not show a strong inverse relationship between DMN and CEN activity. Instead, connectivity analyses indicated suppression within DMN-related regions.

## 1 Introduction

Zen meditation aims to cultivate focused attention and present-moment awareness through non-judgmental observation of internal experience. Rooted in Buddhist Zen traditions, this practice emphasizes self-awareness by minimizing habitual judgments and interpretative biases (Yaoi et al., 2009; Suzuki, 2007; Kozasa et al., 2012). Owing to these characteristics, Zen meditation has attracted increasing attention in neuroscience, metacognition, consciousness research, and education (Cahn and Polich, 2006; Kabat-Zinn et al., 1992; Tan, 2012).

Accumulating evidence suggests that Zen meditation may contribute to psychological well-being by alleviating stress and anxiety and by enhancing emotional regulation. These effects are not limited to individual mental health but extend to social functioning, including improved empathy, comprehension, and interpersonal communication (Tang et al., 2015; Jha et al., 2007; Park and Pinel, 2023; Saini et al., 2021). By fostering calm judgment and reducing emotionally biased decision-making, Zen practice may also mitigate feelings of loneliness and social disconnection.

From a cognitive perspective, Zen meditation can be characterized as attention training that promotes metacognitive awareness. Practitioners are encouraged to observe thoughts and emotions without engagement, typically by sustaining attention on rhythmic breathing and gently redirecting attention when mind-wandering occurs (Suzuki, 2007; Loori, 2006). Such practices share core features with experimental paradigms used to study attentional control and conflict regulation (Hori, 2000; Tang and Posner, 2009; Tang et al., 2012).

Neuroimaging studies have linked mind-wandering to activity within the default mode network (DMN), including the medial prefrontal cortex (MPFC), posterior cingulate cortex (PCC), and precuneus (McVay and Kane, 2009), whereas conflict monitoring and cognitive control are associated with regions such as the anterior cingulate cortex (ACC) and dorsolateral prefrontal cortex (DLPFC) (Atkin et al., 2011). Importantly, evidence regarding DMN engagement during meditation is mixed: while some studies report altered or increased DMN activity in novice meditators (Jang et al., 2011), others suggest DMN suppression and enhanced functional coupling (Hasenkamp et al., 2012; Sorella et al., 2025) between self-monitoring and control-related regions in experienced practitioners (Brewer et al., 2011; Pagnoni et al., 2008).

Despite growing interest, the neural mechanisms underlying Zen meditation—particularly under cognitively demanding or conflict-related conditions—remain incompletely understood (Van Dam et al., 2017). The present study addresses this gap by examining how Zen meditation modulates attentional control and large-scale brain networks during conflict processing.

Our focus was on brain regions associated with the DMN, particularly the MPFC, which is close to the ACC and precuneus, as well as the CEN, which includes the DLPFC. The overlapping region of the ACC and MPFC is primarily located in the medial frontal area of the brain and is often referred to as the dorsal ACC. This region is particularly involved in attentional control and error detection. Our objective was to investigate the responses of these areas to different Stroop test scenarios (congruent vs. incongruent) by examining differences in signal change. The MPFC, DLPFC, and precuneus were selected as specific functional regions of interest (ROIs) based on findings from prior studies.

### 1.1 Historical overview of Zen training

Becoming a Zen monk requires enduring years of rigorous discipline at a primary Zen temple (institute) in Japan. People enroll in a Zen training institute, dedicating several years, often up to seven, to undergo a rigorous disciplinary curriculum. This tradition has been upheld since Eisai Min-nan established Rinzai-Zen, a major Zen school, in Japan in 1195 (Suzuki, 2007). This institution places a strong emphasis on incorporating ‘koans,’ which are thought-provoking queries that monks reflect upon during ‘zazen’ (meditation in a seated, silent posture) to achieve enlightenment.

An illustration of a ‘koan’ is the concept of the ‘sound of one hand clapping,’ questioning what sound a single hand would produce when clapped, given that the conventional action involves both hands. The work combines intentional physical activity to engage specific brain areas with preparation for meditation, creating a state of the embodied mind (Varela et al., 2017). Rinzai-Zen, with its ‘koan’ practice, is often seen as a psychological approach for metacognition, however, it is religious in nature (Austin, 2013; Suzuki, 2007; Loori, 2006; Hori, 2000). Metacognition refers to the process of thinking about one’s own thinking and it involves being aware or understanding one’s cognitive processes and strategies. The ‘koan’ study is an intricate process that explores the relationship between Zen masters and their trainee monks for metacognition (Hori, 2000). This practice, rooted in Rinzai-Zen, has played a significant role in shaping the distinctive aspects of traditional Japanese Zen culture and arts, including the tea ceremony, garden design, painting, calligraphy, and architecture (Suzuki, 2007). All of these can be regarded as traditional art forms that express meditation in a spiritual way.

Here, instead of ‘koan’, we provided the Zen monks a challenging conflict task, the Stroop color task (Stroop, 1935), and explored how the monks overcome the conflict compared with subjects without meditation experience. The Stroop effect refers to a delay in responding to stimuli when there is a discrepancy between the meaning of a word and the color in which it is written. For example, saying the color of the word “green” when it is written in red ink. This mix-up causes longer reaction times and more errors (Stroop, 1935). Previous studies on meditation have certain limitations, as they have not incorporated challenging tasks, such as the Stroop task, to replace a well-crafted ‘koan’ (Hori, 2000). Additionally, much of the existing data only explores the effects of short-term meditation training, lacking insights from highly experienced Zen monks practicing over an extended period.

### 1.2 Central executive network (CEN) and default mode network (DMN)

It seems that the CEN and DMN operate as a seesaw system, sharing attentional resources with each other (Koshino et al., 2011). Mind wandering (based on the DMN) tends to decrease when we engage in challenging, externally focused, resource-demanding tasks, which are typically associated with the activation of the CEN (Kane and McVay, 2012). Mind-wandering occurs when one engages in a stream of thoughts, moving from one topic to another unintentionally, typically during moments of distraction. There is a suggestion that mind-wandering is associated with mentalization, social isolation (Spreng et al., 2020), and inner speech (Fenyhough, 2016), all of which involve the operations of the DMN. Mental relaxation refers to a low-stress, low-effort mental state characterized by reduced cognitive and emotional tension, whereas mind-wandering refers to a shift of attention away from the current task toward internally generated thoughts. Although these states may co-occur, they are conceptually and functionally distinct and do not necessarily arise simultaneously (Menon, 2023). They overlap sometimes, but not always.

On the other hand, activities that require focused attention, such as Zen meditation, partly rely on working memory within the CEN. While there are substantial variations in working memory among individuals (Minamoto et al., 2017), those with lower working memory capacity tend to experience more frequent instances of mind-wandering and commit more errors on incongruent trials in the Stroop task compared to those with higher capacity (Kane and McVay, 2012). This tendency is often linked to deficient attention control (Pardo et al., 1990).

When our minds enter a task-free resting state, allowing for distractive thoughts, it is akin to gently activating the medial prefrontal cortex (MPFC)/ACC of the DMN (Koshino et al., 2013). How does attention work in the prefrontal cortex (PFC) during tasks involving conflict, such as the Stroop task? The role of the PFC, specifically the lateral PFC near the dorsolateral prefrontal cortex (DLPFC) and dorsal anterior cingulate cortex (ACC), is likely related to the shifting of attention during the Stroop task (Vendrell et al., 1995). In incongruent trials, there is a need for rapid attention shifting within the CEN.

In contrast, the DMN exhibits significantly reduced neural activation during meditation compared to periods of mind-wandering (Scheibner et al., 2017), supporting the view that meditation is associated with the suppression of DMN overactivity, with attention serving as a key regulatory function.

During meditation, practitioners must regulate the heightened DMN activity triggered by mind-wandering, and this regulation is accomplished through the attentional control mechanisms of the CEN, helping maintain the meditative state at a balanced and moderate level.

DMN caused by mind-wandering and this is achieved by the attention control mechanism of the CEN, ensuring that the meditative state remains at a balanced and moderate level. The CEN, specifically the DLPFC, modulates the activity of the MPFC (Curtin et al., 2019).

During attention-demanding cognitive tasks, the activity of the CEN increases, allowing the DMN to become more inactive and improve task performance by utilizing attentional resources. This shift is associated with a decrease in distraction, aligning with the need for focused attention during such tasks. Previous research suggests that tasks involving conflict activate the CEN (Brewer et al., 2011; Garrison et al., 2015), while, in contrast, meditation tasks are likely to activate the DMN (Taylor et al., 2013). However, this issue is still under investigation and remains unresolved, as the coactivation of the CEN and DMN has been observed during task preparation (Koshino et al., 2013). When dealing with conflict-related tasks, the brain’s DLPFC plays a crucial role in focusing attention within the CEN which becomes active under Stroop’s incongruent trials (Pardo et al., 1990; Vendrell et al., 1995). Here, we could hypothesize that Zen meditation, known for its concentration, is thought to direct attention through the DLPFC in the CEN and inhibit overactivation in mind-wandering in the MPFC within the DMN.

Expert meditators seem to handle challenging tasks more efficiently by maintaining lower levels of brain activity through skilled attention control, a feat not as pronounced in individuals without meditation experience (Bailey et al., 2020; Brefczynski-Lewis et al., 2007; Kozasa et al., 2012).

In a recent brain imaging study of meditator using fMRI indicated that the MPFC, PCC and precuneus, which are key regions of the DMN, remained active during episodes of mind-wandering (Hasenkamp et al., 2012).

Austin (2016) suggests that Zen meditation involves a three-step working memory process based on the CEN. Initially, focused attention is maintained on each breath (CEN). Subsequently, attention may wander, leading to discursive distraction (DMN). Finally, the CEN recollects attention, redirecting it to the original focus (Austin, 2016). This means that the CEN and DMN work together in sequence (Koshino et al., 2013), with the Salience Network (SN) detecting distraction or mind-wandering. The SN, involving the anterior insula and ACC, helps shift between DMN and CEN to bring attention back to meditation (see Figure 6) (Menon, 2011). Zhang et al. (2021) reported increased resting-state functional connectivity among the PCC, the dorsal attention network (DAN), the superior parietal lobule (SPL), and the DMN following a two-month focused attention meditation training in ten novice meditators, a population comparable to our control participants. Given that the SPL is a key node of the frontoparietal control network (CEN) and functionally interacts with the DLPFC, these findings suggest that even a simple practice such as counting one’s breath can enhance functional connectivity both within and across large-scale brain networks, particularly the DMN and CEN. This pattern is consistent with the notion that focused attention meditation may facilitate switching between mind-wandering and task-focused states, as well as the maintenance of attention once an attentive state is established. Meditation practice is associated with brain regions overlapping with the DMN, the SN, and the CEN, and we hypothesized that intrinsic functional connectivity across these networks is associated with meditation.

So, the SN probably guides the shift from the DMN to CEN systems, steering attention toward meditation (Seeley et al., 2007; Nekovarova et al., 2014; Uddin, 2015). The DMN is active during mind-wandering, often occurring intermittently during sustained attention tasks, while the task-positive network (CEN) supports various attention tasks. Hasenkamp et al. (2012) proposed a four-stage cognitive cycle (mind-wandering, awareness of mind-wandering, shifting attention, and sustained attention) to explain cognitive fluctuations between mind-wandering and attention during focused attention meditation.

### 1.3 Attentional control

To see the underlying brain mechanism of conflict resolution (the Stroop task), it is important to look at the conflict between the more automatic response corresponding to the reading of the word text, and the less automatic, task-prescribed response corresponding to the “ink color” of the word fonts. Attention control is intriguing for its ability to adapt to specific tasks, such as the Stroop task. It enables swift, accurate responses, especially when handling incongruent elements, like choosing the correct color name despite a mismatch with the written text.

The process behind such adaptability, referred to as cognitive control, has been the focus of conflict monitoring in working memory (Botvinick et al., 1999, 2001; Osaka et al., 2007; Raz and Buhle, 2006). Specifically, the primary attention control system of working memory based on the CEN (attention shifting and inhibition) seems critical in the incongruent condition. The CEN network anchored in the DLPFC plays a vital role in attention control. However, very little is known about how the intervention of control processes works in Zen meditation. A report suggested that two executive functions—attention shifting for task management and the inhibition of prepotent responses under a cognitive conflict play a critical role (MacDonald III et al., 2000; Bush et al., 2000; Cohen et al., 2000). Neuroimaging studies have demonstrated that the ACC and the DLPFC are the neural substrates responsible for attention shifting and response inhibition in the Stroop task. The cooperative role of the ACC and DLPFC in attention shifting and inhibition has been widely reported (Bush et al., 2000; Cohen et al., 2000). The ACC is likely involved in metacognitive monitoring and conflict detection and, when such conflict is detected, sends this information to the DLPFC where the conflict is resolved (Kondo et al., 2004; Shenhav et al., 2016). In experienced meditators, short-term meditation practice might lead to reduced activation in neural systems involved in attention regulation, which could be associated with customized and semi-automatized performance in tasks requiring sustained attention (Tang and Posner, 2009).

Using fMRI, here, we showed how professional monk meditators and control participants complete the Stroop task with sustained attention to inhibit responses under a cognitive conflict and how well the monk meditators control attention to prevent wandering during meditation. We hypothesized that experienced monks, due to long-term training at the institute, use the DLPFC in the CEN to suppress conflicting information, and that Zen meditation reduces the activity of the DMN due to distraction, particularly in the MPFC and ACC, leading to heightened awareness. Additionally, this part of the brain may prevent overactivity in the MPFC/ACC and precuneus within the DMN, which may be linked to mind-wandering during meditation.

## 2 Materials and methods

### 2.1 Participants

Nineteen Zen monks (mean age = 39.26 ± 5.91 years) and 18 age- and education-matched (mean education years = 24 ± 2 years; all participants have graduated from university undergraduate programs) healthy male volunteers (mean age = 38.83 ± 6.35 years) were recruited for the study. All Zen monks had undergone training at the professional Institute (Senmon Dojo) of the Rinzai-Zen sect in Kyoto, with a mean training duration of 7.53 ± 2.50 years. Participants in the control group had not received Zen meditation training. Prior to the experiment, a preliminary trial consisting of three blocks was conducted. All participants reported normal or corrected-to-normal vision. Detailed information about the study was provided to each participant by an experimenter, and informed consent was obtained from each participant. The study protocol was approved by the Ethics Committee of the Advanced Telecommunication Research Institute International (ATR) before the experiment. All procedures were conducted in accordance with the guidelines and regulations of ATR International.

### 2.2 Stimuli and tasks

**Figure 1:**
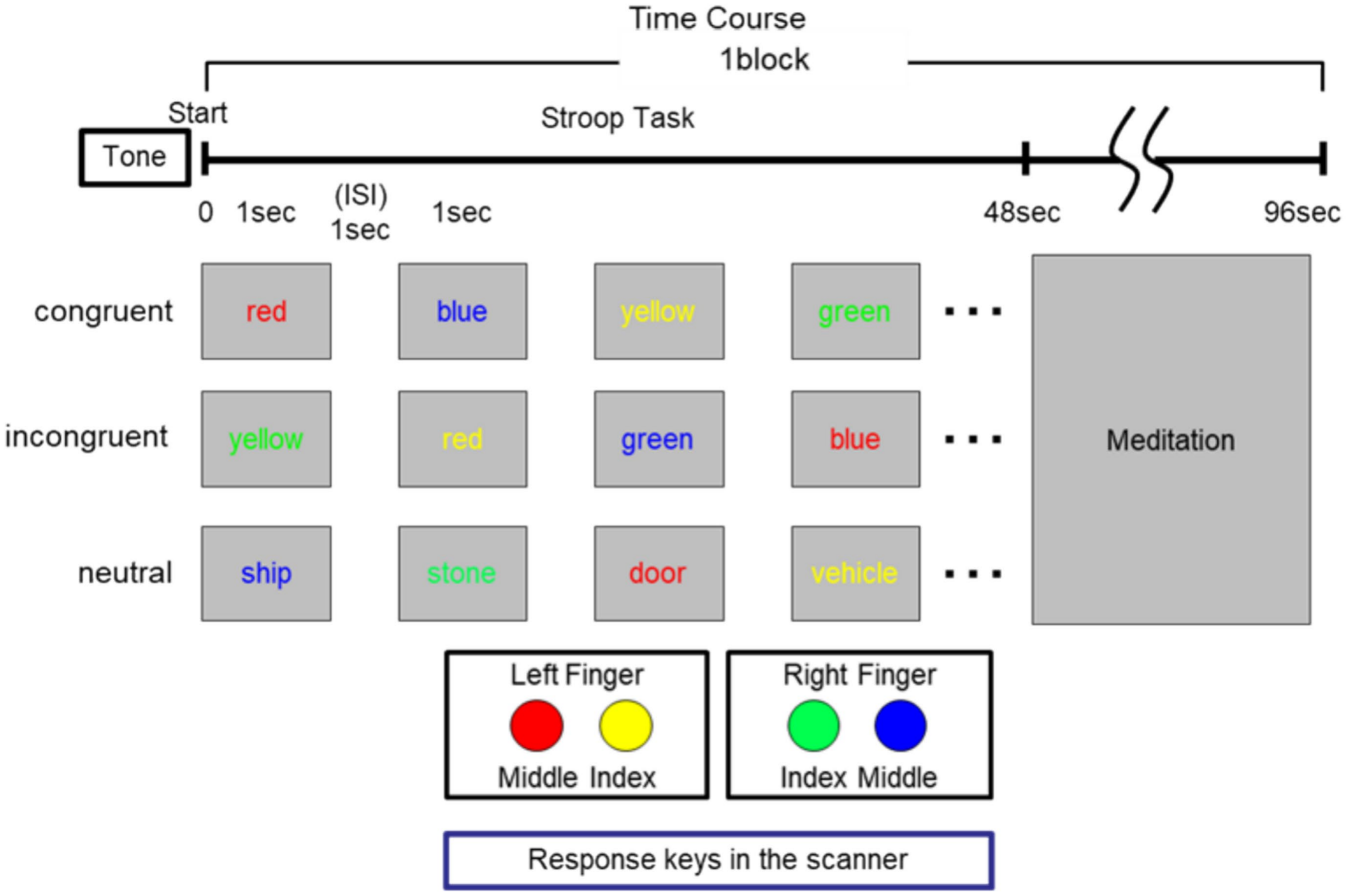
Time progression, order of presentation (congruent, incongruent, and neutral), and positioning of response keys within the scanner.

Using fMRI, we investigated the cognitive effects observed in two groups of subjects exposed to a task inducing cognitive conflict, namely the Stroop color task, during the former half of the block (1–48 s). In this task, participants were required to identify the color in which a word was presented, with the words themselves having congruent (e.g., the word “red” written in red ink) or incongruent (e.g., the word “red” written in green ink). We employed four color words (red, yellow, green, and blue) and four non-color words (stone, door, ship, and vehicle), all represented in Japanese Kanji characters. Each word was printed in one of the four colored inks (red, yellow, green, or blue) (see Figure 1).

Participants underwent a standard Stroop color task (Stroop, 1935) while inside the MRI scanner. They were instructed to identify the ink color of the word while disregarding its verbal meaning. The task comprised three conditions: congruent, incongruent, and neutral. In the congruent condition, the ink color always matched the color name of the word (e.g., the word “red” printed in red ink). Conversely, in the incongruent condition, the ink color conflicted with the color name of the word (e.g., the word “red” printed in green ink). Non-color words were presented in the neutral condition (e.g., the word “stone” printed in green ink). Word stimuli were presented with a visual angle of 3° × 3° through a mirror mounted on a head coil. Participants were tasked with pressing one of four buttons within the scanner, with each button corresponding to a specific color to report (red, yellow, green, or blue) (see Figure 1). Each block lasted 96 s and consisted of two 48-s periods, during which all three task conditions were presented. Participants performed the Stroop trials during the first half of each block. In the latter half, they were instructed to refrain from task performance and to simply count their breath, following a common Japanese meditation instruction used for individuals with or without prior meditation experience. Although the overall meditation instruction was comparable across groups, the meditation condition during the latter half of the block (48–96 s) was not entirely identical: control participants (CTRL) were asked to maintain mental quietude by counting their breath, while Zen monks (MEDT) implicitly followed their habitual, structured meditation practice based on breath counting.

Participants were required to select the response key corresponding color from four options as quickly and accurately as possible using buttons for both hands (Figure 1). After completing the trials, participants entered a 48-s meditation period, which in practice corresponds to a resting period, as shown in Figure 1. During meditation, participants were instructed to close their eyes and focus on maintaining a state of mental quietude without engaging in any thoughts or contemplation. However, whether participants were actually able to maintain such a thought-free state is inherently subjective and difficult to verify or control experimentally. A tone signaled the start of each block, with block order pseudo-randomized. The Stroop blocks were separated by a 48-s meditation period, repeated six times. Stimulus presentation and response recording were controlled using Presentation software (Neurobehavioral Systems, Inc., Albany, CA). We refrained from including instructions on the frequency or nature of mind-wandering during meditation, as we believe such instructions could potentially alter the meditative experience.

### 2.3 Apparatus and procedure

Functional magnetic resonance imaging (fMRI) data were acquired using a 3.0-T MRI scanner (MAGNETOM Verio, Siemens, Munich, Germany). Participants’ head movements were minimized with a forehead strap and comfortable padding. Functional images sensitive to blood oxygen level-dependent (BOLD) contrasts were acquired using a single-shot echo-planar imaging sequence (repetition time (TR) = 2000 ms, echo time (TE) = 30 ms, flip angle = 80°, 64 × 64 at 3-mm in-plane resolution, 4-mm thickness with a 1-mm gap, 30 contiguous oblique axial slices parallel to the AC–PC line), resulting in 1,162 images. Subsequently, anatomical images were obtained for all participants (TR = 2,250 ms, TE = 3 ms, flip angle = 9°, voxel size = 1 × 1 × 1 mm^3^). All data were analyzed using SPM 5 (Wellcome Department of Imaging Neuroscience) on Matlab 7.3 (MathWorks Inc^1^). The first six images were discarded from the analysis to eliminate any non-equilibrium effects of magnetization. All functional images were realigned to correct for head movements, which were less than 3.0 mm within runs. Functional images were normalized and spatially smoothed with an isotropic Gaussian filter (8 mm full-width at half-maximum). Low-frequency noise was removed with high-pass filtering (128 s). For the Stroop task and meditation, we set 48 s functional blocks (congruent, incongruent, neutral and meditation) and modeled with a gamma hemodynamic response function (HRF) that was applied when each block started. Group data were analyzed using a random effects model. Activation areas for all conditions were specified at *p* < 0.05 cluster level FWE corrected for multiple comparisons with the amplitude of voxels surviving at *p* < 0.001 uncorrected across the whole brain. Percentage signal change analyses of the regions of interest (ROIs) were performed using STATISTICA (StatSoft, Inc., Salsa, OK).

#### 2.3.1 Connectivity analysis

For the connectivity analysis, we utilized CONN (CONN Toolbox Manual^2^). After data preprocessing, we employed CONN’s default denoising pipeline to reduce the residual noise affecting the blood oxygenation level-dependent signal. We examined the differences between the meditator group and the control group in incongruent, congruent, neutral, and meditation conditions using CONN. An anatomical component-based noise correction procedure was employed. Using CONN’s ROI-to-ROI analyses, we estimated functional connectivity, focusing on connections within the three primary resting state networks: the CEN, DMN, and Salience Network (SN). Additionally, we examined network connections involving brain regions activated during meditation. Nineteen default ROIs from CONN’s ICA analyses of the Human Connectome Project dataset were selected. These included ROIs from the ‘networks.DefaultMode’ (MPFC, LP(lateral parietal;left), LP(right), PCC), ‘networks.DorsalAttention’ (FEF(frontal eye fields;left), FEF(right), IPS(intraparietal sulcus;left), IPS(right)), ‘networks.FrontoParietal’ (LPFC(lateral prefrontal cortex;left), LPFC(right), PPC(left), PPC(right)), and ‘networks.Salience’ (ACC, AInsula(anterior insula;left), AInsula(right), RPFC(rostal prefrontal cortex;left), RPFC(right), SMG(spramarginal gyrus;left), SMG(right)) groups. The ‘networks.DefaultMode’ ROIs represent typical brain areas associated with the resting state network, identified through functional connectivity analysis of resting state data. ROIs from the ‘networks.DorsalAttention’ and ‘networks.FrontoParietal’ groups constitute the CEN, while those from the ‘networks.Salience’ group are implicated in network switching.

## 3 Results

### 3.1 Behavioral data

**Figure 2:**
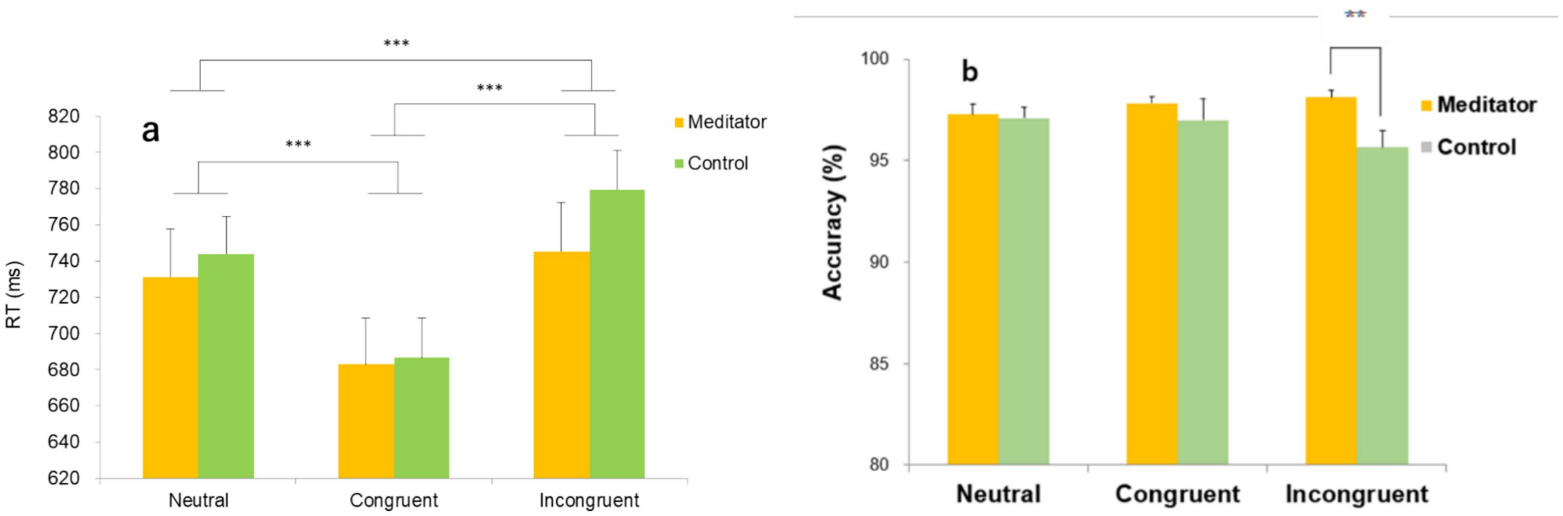
Response time (ms) **(a)** and accuracy rate (%) **(b)** vary according to the condition for both the meditator (MEDT) and control (CTRL) groups. Error bars represent the standard error of the mean (SEM). *p < 0.10, **p < 0.05, ***p < 0.001.

The behavioral results were assessed using response times (in ms) and the accuracy rate (% correct) (Figures 2a,b, respectively).

We conducted an exploratory two-way analysis of variance (ANOVA) with group (meditator [MEDT] vs. control [CTRL]) as a between-subject factor and condition (neutral, congruent, incongruent) as a within-subject factor. All participants responded to all three conditions.

For response time, a two-way ANOVA (group × condition) revealed a significant main effect of condition [*F*(2,70) = 63.76, *p* = 0.0001, η2p = 0.646], whereas the main effect of group was not significant [*F*(1,35) = 0.24, ns, η2p = 0.07]. The group × condition interaction did not reach conventional statistical significance but showed a trend toward significance [*F*(2,70) = 2.52, *p* = 0.088, η2*p* = 0.067].

Given this trend-level interaction, we conducted *post hoc* multiple comparisons using Shaffer’s modified sequentially rejective Bonferroni procedure, which provides greater statistical power while appropriately controlling the family-wise error rate. These analyses revealed a significant difference between the neutral and incongruent conditions in the CTRL group (*p* < 0.005), whereas no such difference was observed in the MEDT group (Figure 2a).

Figure 2b shows the correct response rates across the neutral, congruent, and incongruent conditions for the MEDT and CTRL groups. A two-way ANOVA examining the effects of condition and group revealed no significant main effects of condition [*F*(2,70) = 0.56, *p* = 0.58, η2p = 0.016] or group [*F*(1,35) = 2.25, *p* = 0.14, η2p = 0.06]. However, the group × condition interaction did not reach conventional significance but showed a trend toward significance [*F*(2,70) = 2.94, *p* = 0.06, η2p = 0.08].

Given this trend-level interaction, we conducted exploratory *post hoc* multiple comparisons using Shaffer’s modified sequentially rejective Bonferroni procedure to further characterize potential group differences while controlling for multiple comparisons. These analyses indicated a significant difference between the MEDT and CTRL groups (MEDT > CTRL) only in the incongruent condition (*p* < 0.05).

The longer response times and increased response errors predominantly observed in the incongruent condition imply cognitive conflict arising from lexical mismatches in the task. This conflict is believed to be a primary driver for the engagement of attentional control mechanisms (Austin, 2016; Botvinick et al., 1999; Botvinick et al., 2001).

In the control (CTRL) group, the response time was slower in the incongruent condition compared to the neutral condition due to the color mismatch effect inherent in the Stroop task. Conversely, Zen meditators (MEDT) did not exhibit a response time difference under the same conditions, indicating reduced conflict among meditators (Figure 2a). The CTRL group also showed a trend towards greater standard Stroop effect in reaction time than the MEDT group (Figure 2a). Moreover, the MEDT group showed a numerically higher rate of correct responses (% accuracy) than the CTRL group during the incongruent condition (Figure 2b), but the difference was not statistically significant. These behavioral findings suggest that meditators may effectively suppress cognitive conflict stemming from color mismatch compared to the control group. The study underscores the significant role of attentional control prompted by cognitive conflicts, such as those arising from mismatches between color names and their printed colors.

### 3.2 fMRI data

**Figure 3:**
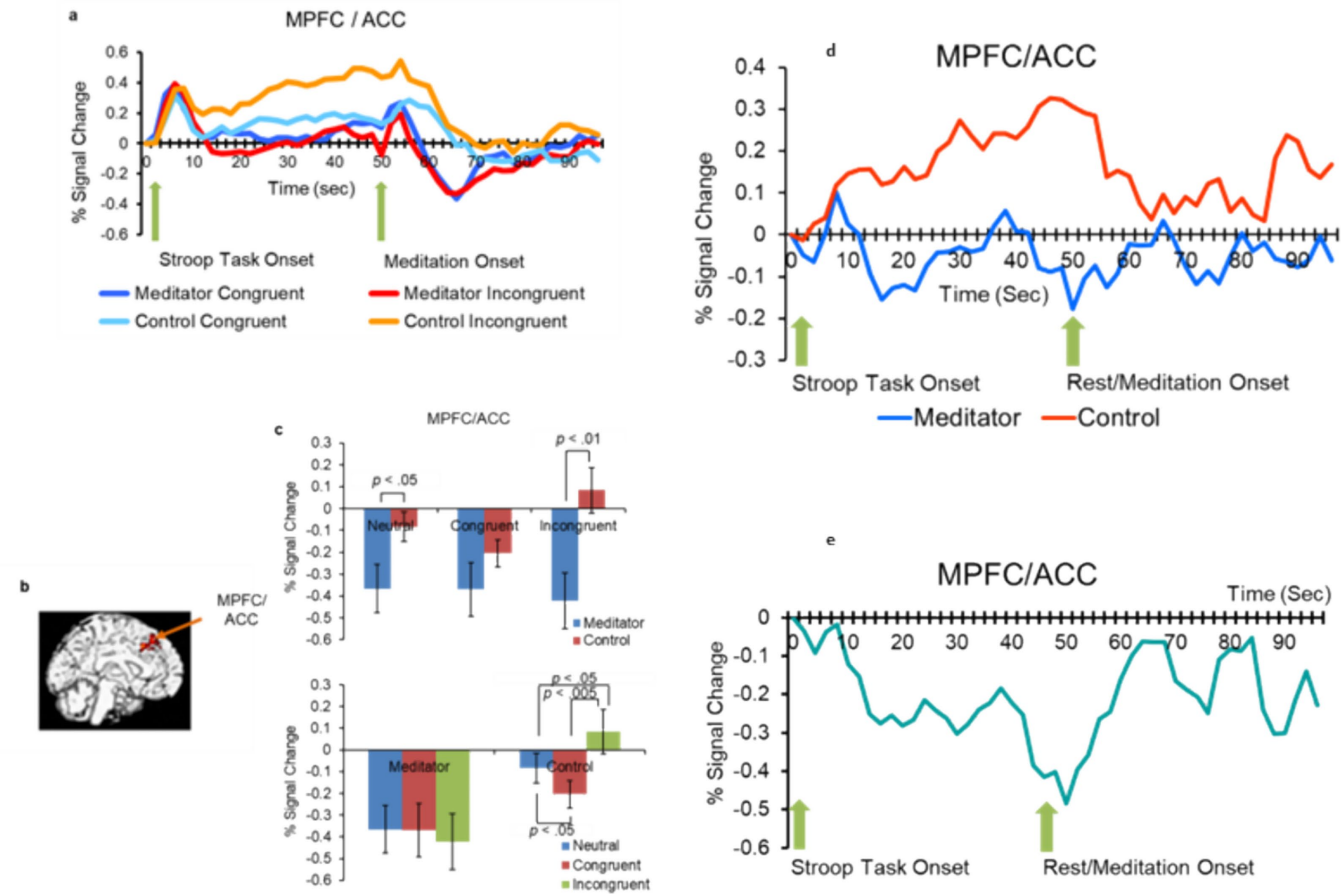
**(a)** Percentage of signal change over time following the onset of the Stroop color task (first arrow) and meditation (second arrow, 48 s after the Stroop color task onset). Activation patterns in the broader MPFC and ACC under incongruent, congruent, and neutral conditions for both the meditation (MEDT) and control (CTRL) groups. **(b)** Emphasis is on the MPFC/ACC and a segment of the ACC within the medial brain. **(c)** Signal change is analyzed with respect to conditions (incongruent, congruent, and neutral) and groups (MEDT and CTRL), with error bars representing the standard error of the mean (SEM). **(d)** The average difference between incongruent and congruent conditions is plotted for the MEDT group in blue and the CTRL group in red. **(e)** Disparities (meditator-control) between the blue (meditator) and red (control) lines in **(d)** are illustrated in graph **(e)**.

**Figure 4:**
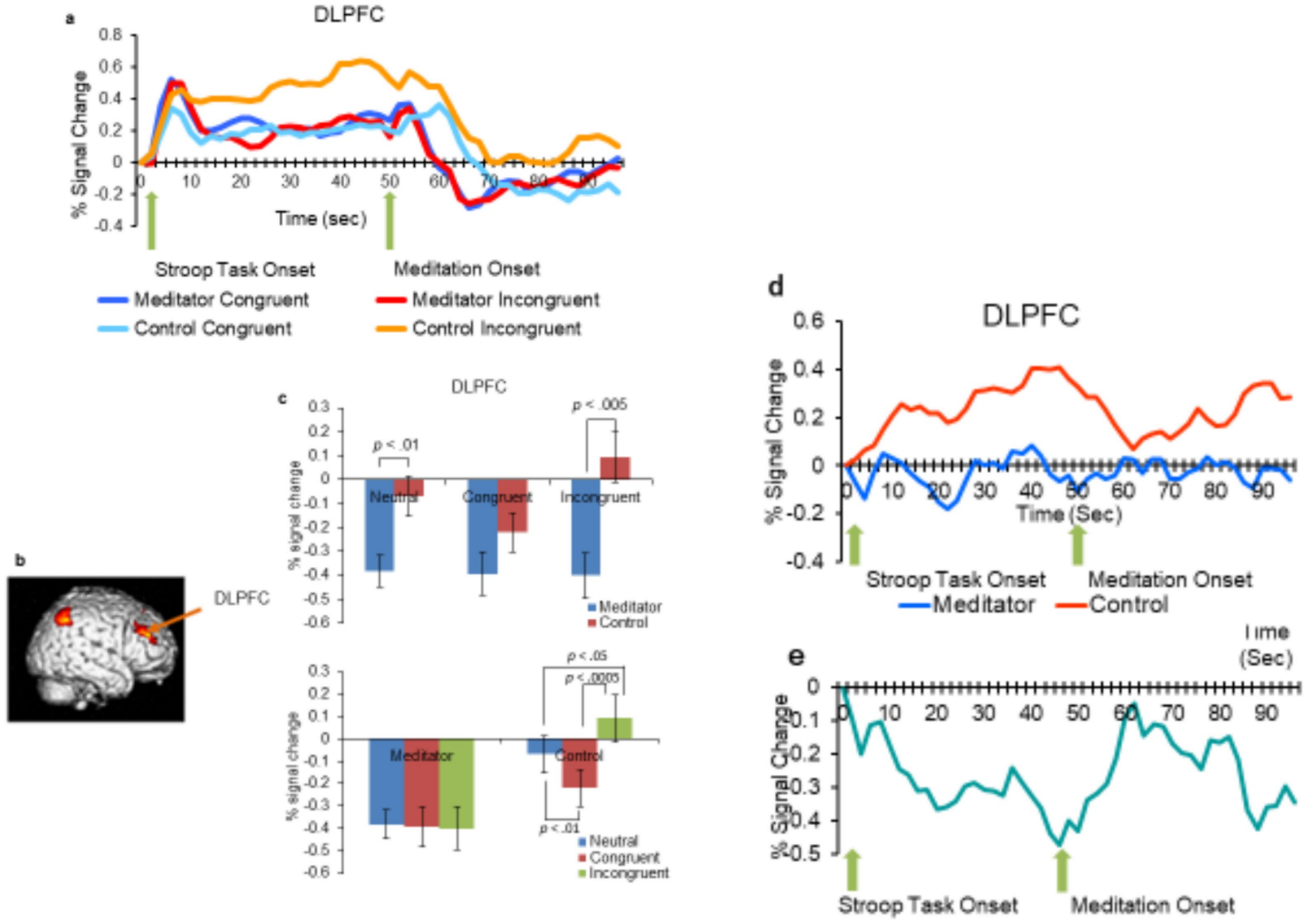
**(a)** Percentage of signal change over time following the onset of the Stroop color task (first arrow) and meditation (second arrow, 48 s after the Stroop color task onset). Activation patterns in the DLPFC under incongruent, congruent, and neutral conditions for both the MEDT and CTRL groups. **(b)** Emphasis is placed on the DLPFC within the lateral brain. **(c)** Signal change is analyzed with respect to conditions (incongruent, congruent, and neutral) and groups (MEDT and CTRL), with error bars representing the standard error of the mean (SEM). **(d)** The average difference between incongruent and congruent conditions is plotted for the MEDT group in blue and the CTRL group in red. **(e)** Disparities (meditator-control) between the blue and red lines in **(d)** are illustrated in graphs **(e)**.

**Figure 5:**
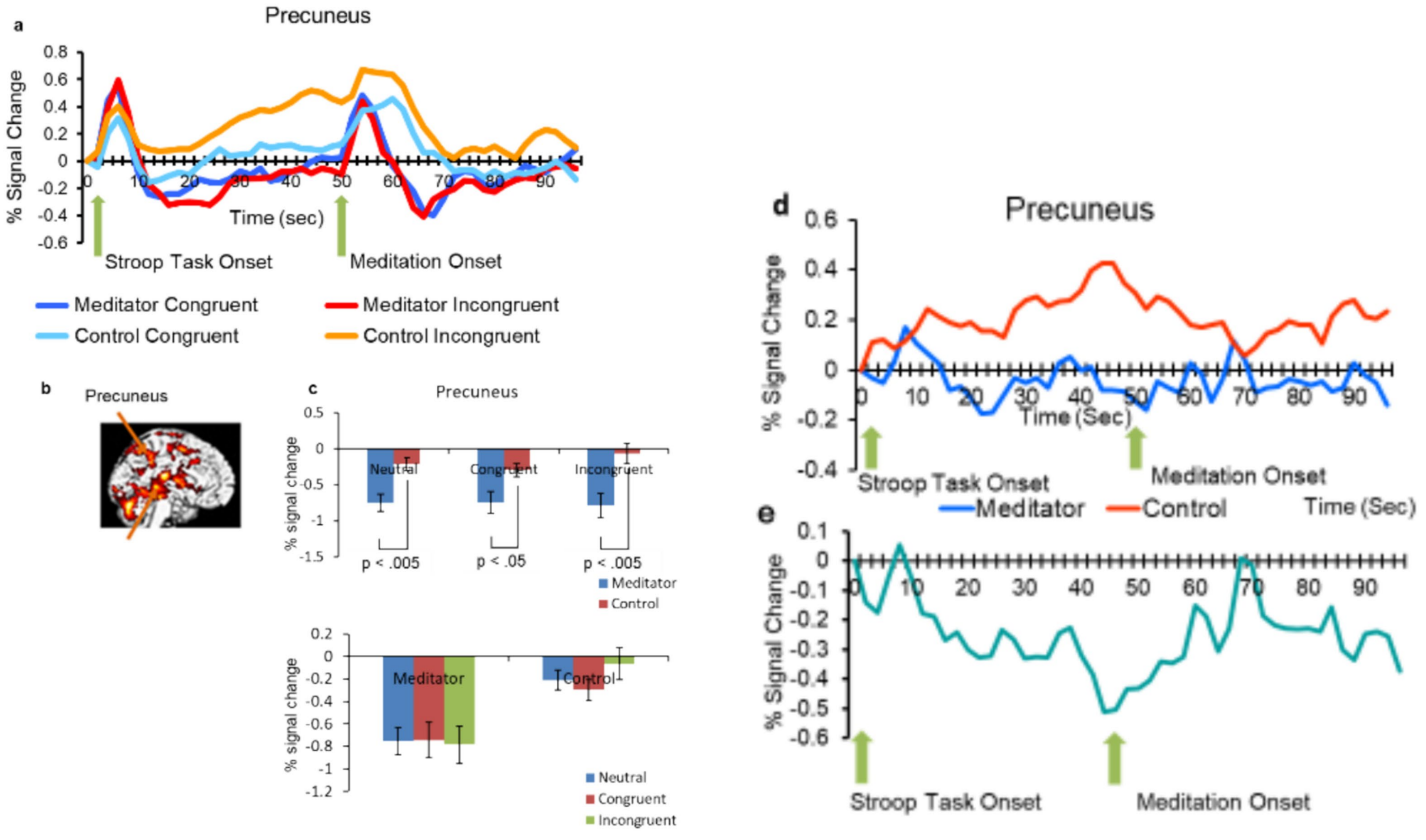
**(a)** Percentage of signal change over time following the onset of the Stroop color task (first arrow) and meditation (second arrow, 48 s after the Stroop color task onset). Activation patterns in the precuneus within the brain under incongruent, congruent, and neutral conditions for both the MEDT and CTRL groups. **(b)** Emphasis is placed on the precuneus. **(c)** Signal changes in the precuneus for both the MEDT and CTRL groups, with error bars representing the standard error of the mean (SEM). **(d)** The mean difference between incongruent and congruent conditions is plotted for the MEDT group in blue and the CTRL group in red. **(e)** The disparity (meditator-control) between the blue and red lines in **(d)** is depicted in graphs **(e)**.

Our focus was on brain regions associated with the DMN, particularly the MPFC, which is close to the ACC and precuneus, as well as the CEN, which includes the DLPFC. The overlapping region of the ACC and MPFC is primarily located in the medial frontal area of the brain and is often referred to as the dorsal ACC. This region is thought to be particularly involved in attentional control and error detection. Our objective was to investigate the responses of these areas to different Stroop test scenarios (congruent vs. incongruent) by examining differences in signal change. We selected specific functional Regions of Interest (ROIs) for the MPFC (10, 32, 38), DLPFC (40, 36, 36), and precuneus (0, –46, 48), informed by insights from prior research (Kozasa et al., 2012; Cohen et al., 2000; Kondo et al., 2004; Shenhav et al., 2016; Koshino et al., 2013). Spheres were generated for clusters using SPM5, each with a 5 mm radius to ensure comprehensive coverage, and MarsBar was utilized for this purpose (Brett et al., 2002). From the ROI-based analysis, during a Stroop task, the meditation group (MEDT) exhibited significantly lower brain activity compared to the control group (CTRL) across various regions including the DLPFC (Figures 3–5).

Figures 3–5 depict the averaged percentage changes in BOLD signal over time following the onset of the Stroop task and meditation (48 s after the Stroop task onset). Activations in the MPFC/ACC (Figures 3a,b), DLPFC (Figures 4a,b), and precuneus (Figures 5a,b) are presented under incongruent, congruent, and neutral conditions for both groups. Additionally, the difference in signal change between the incongruent and congruent conditions is illustrated for the MEDT (blue) and CTRL (red) groups in (c), along with their difference plot (e) (Figures 3–5).

#### 3.2.1 MPFC/ACC

Figure 3 depicts the signal fluctuations in the MPFC/ACC throughout the Stroop conflict task (from 0 to 48 s) and meditation (from 49 to 96 s). Notably, there was a rapid peak in the signal 5 s after the initiation of the Stroop task (first arrow), followed by a sharp decline. This may simply reflect the brain response to the saliency of the event representing the beginning of a new task condition (Stroop or Meditation). Likewise, after the onset of meditation (second arrow), there was a rapid increase in the signal at 2 s, followed by a decline 4–5 s later. These fluctuations were particularly pronounced in the meditation group compared to the control group.

We conducted a two-way ANOVA, which revealed significant main effects of group [*F*(1, 35) = 4.92, *p* = 0.033, η2p = 0.123] and condition [*F*(2, 70) = 4.19, *p* = 0.019, η2p = 0.107] on signal change. Additionally, the interaction between groups and conditions was found to be significant [*F*(2,70) = 9.10, *p* = 0.0003, η2p = 0.206]. *Post hoc* multiple comparisons using Shaffer’s modified sequentially rejective Bonferroni procedure revealed that the CTRL group displayed significant differences between the neutral and incongruent conditions (*p* < 0.05).

#### 3.2.2 DLPFC

In general, similar patterns were observed in the Dorsolateral Prefrontal Cortex (DLPFC) (Figure 4). A notable increase in signal change occurred 4–3 s after the onset of the Stroop task, followed by a significant decrease 5 s after the initiation of meditation, particularly evident in the MEDT group compared to the CTRL group. As we hypothesized, experienced meditators, due to their long-term training at the institute, tend to use the DLPFC in the CEN to suppress distracting information in tasks such as the Stroop task and to prevent activity in the DMN, which is associated with distraction and mind-wandering during meditation period.

In a study, the signal change in the incongruent condition for the CTRL group remained elevated until the onset of meditation, indicating significant conflict compared to other conditions. We conducted a two-way ANOVA. The DLPFC exhibited main effects of both groups [*F*(1,35) = 7.39, *p* = 0.010. η2p = 0.174] and conditions [*F*(2,70) = 8.42, *p* = 0.0005, η2p = 0.194] on signal change, with a significant interaction [*F*(2,70) = 9.25, *p* = 0.0003, η2p = 0.209]. *Post hoc* multiple comparisons using Shaffer’s modified sequentially rejective Bonferroni procedure were conducted. The CTRL group displayed significant differences between the neutral and incongruent conditions (*p* < 0.05).

#### 3.2.3 Precuneus

The scan pattern in the precuneus closely resembled that of the DLPFC, showing a notable spike in signal change 3 s after the onset of the Stroop task. Situated in the posterior parietal lobe, the precuneus is a key region within the DMN that shows activation during rest and is not focused on external tasks. It is heavily involved in mind-wandering and mentalizing. The precuneus helps integrate information across different networks, as well as connects with other DMN regions, such as the MPFC and PCC.

We conducted a two-way ANOVA and found a significant main effect of group was observed [*F*(1,35) = 9.82, *p* = 0.005, η2p = 0.219], whereas the main effect of condition was not significant [*F*(2,70) = 1.40, ns, η2p = 0.039]. The group × condition interaction did not reach conventional statistical significance but showed a trend toward significance [*F*(2,70) = 2.95, *p* = 0.058, η2p = 0.078].

Exploratory *post hoc* analyses indicated group-related differences in signal change between the MEDT and CTRL groups in the neutral (*p* < 0.005), congruent (*p* < 0.05), and incongruent (p < 0.005) conditions. Within the MEDT group, no statistically significant differences among conditions were observed, except for a difference between the congruent and incongruent conditions, as shown in Figure 5. Specifically, the MEDT group displayed lower signal changes compared to the CTRL group in the precuneus, insular, and bilateral hippocampus suggesting potential effects of meditation on these brain regions. Specifically, insular activation may be related to the SN. In Figures 3a, 4b, 5a, only the Congruent and Incongruent conditions are shown, because the supplementary Neutral condition showed response patterns similar to the Congruent condition. Brain regions collaborate, and their correlations reveal extensive networks. Key networks include the CEN, DMN and Salience Network (SN). The CEN connects the DLPFC to the posterior parietal lobes, indicating its involvement in cognitive attention control. During Zen meditation, the CEN likely engages in sustained attention, given the strong association of the DLPFC with this cognitive function. The DMN primarily links the ventral MPFC and PCC, encompassing areas such as the angular gyrus and medial temporal lobe. It operates in contrast to the CEN, with one network being active while the other is inhibited. In a study involving 12 experienced Zen meditators and 12 meditation-naive individuals, alterations in functional connectivity were observed in the DMN, CEN, and SN, indicating meditation-induced changes in these neural networks (Kemmer et al., 2015).

**Figure 6:**
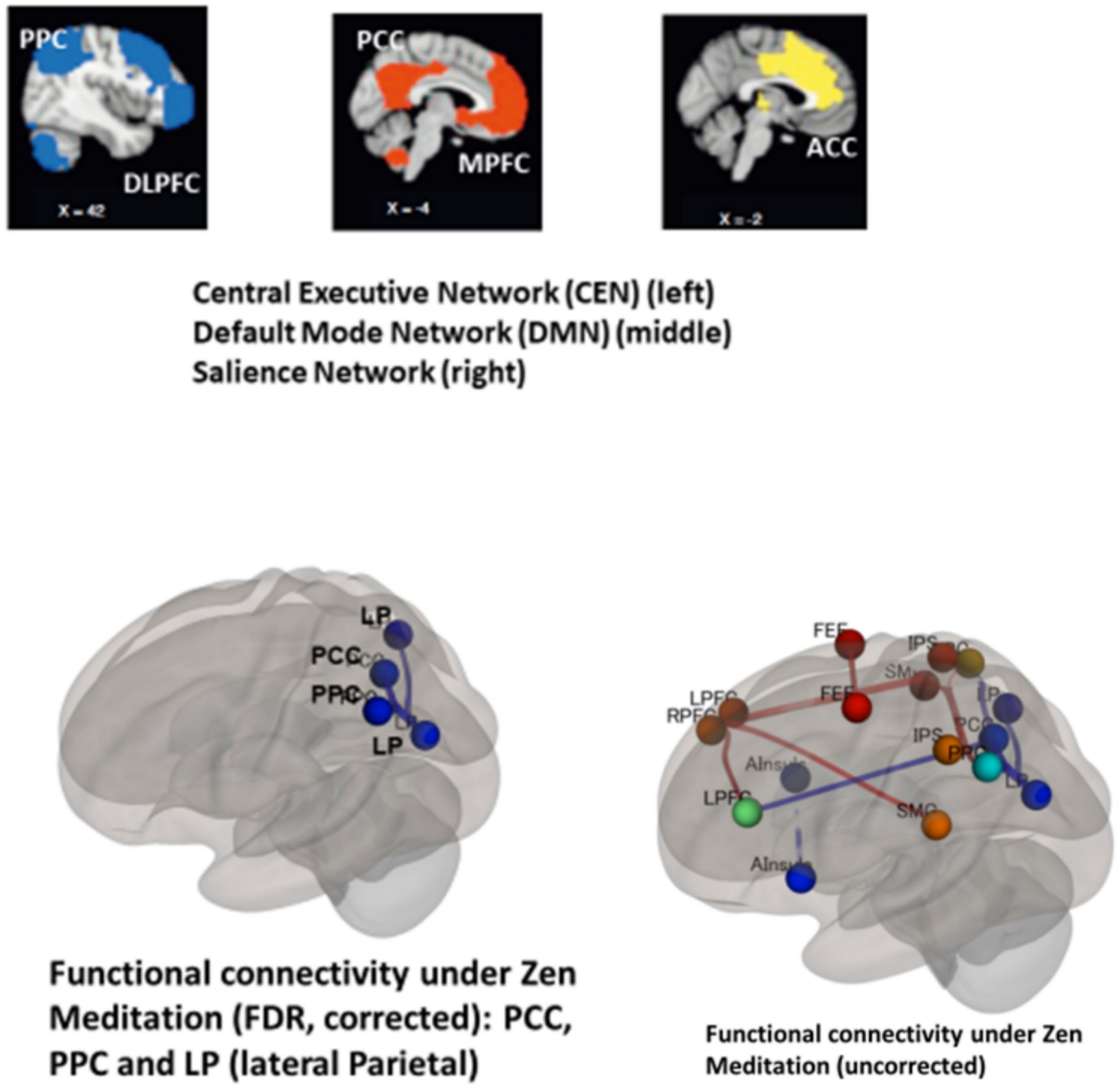
(Upper panel) Illustrated are the Central Executive Network (CEN), which connects the DLPFC and posterior parietal cortex (PPC), Default Mode Network (DMN) linking the Medial Prefrontal Cortex (MPFC) and Posterior Cingulate Cortex (PCC), and the Salience Network (SN) within the brain (partially adapted from Menon, 2011). (Lower panel) Depicted is the functional network connectivity during Zen meditation (meditation-control), in which participants meditated for 48 s with closed eyes and without conflicting task. The network representation employs colored lines, with blue denoting inhibitory connections and red indicating excitatory connections.

When synchronization or correlation of brain activity is observed between brain regions, it is referred to as functional connectivity. In this study, we examined the differences between the MEDT group and the CTRL group under the conditions of incongruent, congruent, neutral, and meditation using CONN (aCompCor; Whitfield-Gabrieli and Nieto-Castanon, 2012; Osaka et al., 2021). As a result, a difference between the MEDT and CTRL groups was observed only under the meditation condition (Figure 6, lower left panel).

The functional network connectivity during Zen meditation (meditation-control) showed significant group disparities, while group differences in the congruent-control and incongruent-control conditions showed no significant disparities. Inhibitory connections were noted among the PCC within the DMN, the PPC within the CEN, and the lateral parietal (LP) cortex (ROI-to-ROI analysis; FDR corrected, *p* < 0.05: left panel). Note that attention control in the PPC appears to be suppressed in the MEDT group. Additionally, under meditation conditions, both inhibitory and excitatory connections were observed among the lateral and medial cortices (uncorrected for multiple comparisons, *p* < 0.05: Figure 6 right). Blue and red lines represent inhibitory and excitatory connections, respectively. Notably, inhibitory connections between the right PPC-ACC and between the anterior insula and supramarginal gyrus were observed under incongruent conditions (uncorrected for multiple comparisons, *p* < 0.05, not shown here).

## 4 Discussion

### 4.1 Behavioral data

The longer response times and increased response errors predominantly observed in the incongruent condition imply cognitive conflict arising from lexical mismatches in the task. This conflict is believed to be a primary driver for the engagement of attentional control mechanisms (Austin, 2016; Botvinick et al., 1999; Botvinick et al., 2001). The CTRL group tended to show longer response times in the incongruent condition than in the neutral condition, consistent with Stroop-related conflict, whereas no statistically significant difference between these conditions was observed in the MEDT group. Participants without experience in Zen meditation tended to show longer response times under conflict conditions (neutral vs. incongruent). With respect to correct response rates, the MEDT group showed a numerically higher percentage of accuracy in the incongruent condition.

Taken together, these behavioral findings may suggest that meditation practice is associated with a greater ability to manage or attenuate cognitive conflict arising from color interference, relative to the CTRL group.

According to behavioral data, meditators outperform controls in managing cognitive conflict during incongruent conditions in the Stroop task. This suggests that effective attentional direction helps inhibit verbalization of color names. Meditators exhibit the capacity to disregard the meaning of the color name and concentrate on selecting the ink color in incongruent conditions (Augustinova and Ferrand, 2014). These behavioral findings suggest that meditators may effectively suppress cognitive conflict stemming from color mismatch compared to the control group. The study underscores the significant role of attentional control prompted by cognitive conflicts, such as those arising from mismatches between color names and their printed colors.

### 4.2 fMRI data

fMRI data analysis of signal changes corroborates the behavioral evidence. The figures depict gradual increases in differences in signal changes between the meditator (MEDT) and control (CTRL) groups over time during a Stroop task. Figures 3d–5d suggest consistently lower activation in all examined brain regions in the MEDT group compared to the CTRL group, particularly evident when comparing incongruent and congruent conditions. Additionally, Figures 3e–5e show a trend towards the difference between incongruent and congruent trials for the MEDT and CTRL groups. This suggests that meditation helped the control group recover from conflict (from 48 to 60–70 s), but likely led to disruption or mind-wandering from 60–70 to 96 s.

In Figure 3, the signal change in the MPFC/ACC reaches its peak 3 s after the Stroop task begins, then gradually returns to baseline. Upon the commencement of meditation, another increase occurs 5–7 s later in both groups. Throughout the process, the MEDT group consistently exhibits a lower overall activation decrease compared to the CTRL group. The graph (e) (Figure 3) shows that the difference between meditation and control tends to decrease 8–10 s after the onset of meditation, suggesting that the DLPFC may be strengthening inhibition during meditation.

We observed activation in the dorsal region of the ACC during the Stroop task, confirming previous findings (Roelofs et al., 2006). There was a notable decrease in activation in both regions 6 s after the onset of the Stroop task and approximately 5–7 s after the onset of meditation in the incongruent trials in the meditation group. The reduced activation of the DLPFC and ACC during incongruent trials in the MEDT group suggests that inhibitory control signals may be involved, possibly linked to the ACC’s function as the brain’s error detection and correction mechanism (Botvinick et al., 1999; Botvinick et al., 2001).

Conflict monitoring plays a crucial role in the executive function of the CEN, involving the ACC and DLPFC. The dorsal frontoparietal system within the working memory network is likely involved in conflict monitoring during tasks such as naming colors with incongruent stimuli. The ACC and DLPFC appear to be linked to conflict monitoring, which is essential for sustaining focused attention (Botvinick et al., 2001).

The MPFC/ACC displayed heightened activity when confronted with incongruent stimuli, consistent with its role in performance monitoring (MacDonald III et al., 2000). In a Stroop interference scenario, non-meditators exhibited increased activation in various prefrontal cortex regions, including the DLPFC, inferior frontal gyrus, and superior parietal lobule (Langenecker et al., 2004).

Experienced meditators who underwent 4 weeks of meditation training demonstrated a reduction in episodes of mind-wandering compared to those engaged in control tasks (Austin, 2016, p. 73). This suggests that longer meditation training may have contributed to a decrease in the frequency of mind-wandering among these individuals. Functional connectivity analysis revealed strong coupling among the DLPFC, dorsal ACC, and PCC in meditators, both in baseline states and during meditation, with key nodes of the DMN activated during mind-wandering (Mittner et al., 2014; Brewer et al., 2011). As previously noted, the cognitive cycle model proposed by Hasenkamp et al. (2012) hypothesized that periods of mind-wandering would be associated with the DMN, while the SN and CEN are associated with awareness of mind-wandering and with shifting and sustained attention, respectively. Fluctuations in the current data during meditation could possibly partly be explained by this cycle model.

Experienced meditators exhibit an overall decrease in activation across the MPFC/ACC, DLPFC, and precuneus compared to non-meditators, indicating enhanced sustained attention during meditation (Posner and Petersen, 1990; Langenecker et al., 2004; Brewer et al., 2011). Conversely, non-meditators exhibit greater overall activation in these networks. Individuals with at least 3 years of meditation practice (three times a week) show the same tendency under incongruent conditions (Bailey et al., 2020). Bailey et al. (2020) suggested, based on electroencephalogram data, that experienced meditators display improved response accuracy and reduced neural activation due to increased neural efficiency.

The lower activation observed in the MEDT group is attributed to their prior Zen meditation training acquired during their time at the Zen institute, and this effect appears to persist strongly and persistently. The impact was firmly established and maintained its potency, operating at a subconscious level to counter cognitive conflict (Posner and Petersen, 1990). Hence, it is inferred that training spanning several years has nurtured attention-based executive control (CEN) to mitigate cognitive conflict.

As a key executive component of the CEN, the DLPFC is likely involved in behavioral adjustment and attention shifting (Brewer et al., 2011; Bailey et al., 2020; Lutz et al., 2008; Brefczynski-Lewis et al., 2007; Taylor et al., 2013).

The scan pattern observed in the precuneus, as shown in Figure 5, closely resembles that of the DLPFC, though it deviates somewhat from our prediction. These results suggest that the selected precuneus ROI is unlikely to be part of the DMN; rather, it may belong to a fronto-parietal attentional network, given that its anatomical location is superior to the retrosplenial cortex, which is typically associated with the DMN. Additionally, the precuneus shares connections with the temporoparietal junction and inferior parietal lobule, as well as prefrontal areas linked to the DLPFC. These interconnected regions play a crucial role in cognitive functions, particularly in the executive functions of focusing and shifting attention within the CEN, while also preventing entrapment in cognitive conflict (De Filippi et al., 2022).

Engaging in cognitively demanding tasks that activate the task-positive network (CEN), especially the DLPFC, leads to reduced DMN activity, which is associated with mind-wandering (Menon, 2011; Posner and Petersen, 1990). In the Stroop task, the DLPFC becomes active, while the MPFC shows activation during the preparation phase and deactivation during task execution, indicating complex temporal dynamics (Koshino et al., 2011). This pattern suggests that the DMN and CEN may not always exhibit strict anti-correlation during tasks typically associated with CEN engagement (Bailey et al., 2020).

Overall, the study consistently suggested significantly lower activation in the MEDT group compared to the CTRL group in the MPFC/ACC (DMN), DLPFC (CEN), and precuneus under both the conflict and meditation conditions. This pattern can be partly explained by the free energy principle, according to which the brain suppresses excessive activity arising from distraction and mind-wandering (Solms and Friston, 2018; Solms, 2021; Friston et al., 2006).

This indicates a prevailing trend in the observed results, as illustrated in Figures 3–5. As we hypothesized, monks are likely to utilize the DLPFC to inhibit mind-wandering in specific scenarios and to suppress activity in the DMN, although the current data could not confirm this strongly.

Therefore, the data suggest shared activation of the CEN and DMN due to sustained attention within the CEN.

Monks remain composed under pressure, navigating cognitive conflict with far greater ease than the average person. Their calm demeanor during tasks such as the Stroop task suggests that the practice of Zen meditation enables them to sustain a heightened and consistent level of inhibitory awareness.

As we hypothesized, experienced meditators, due to their long-term training at the institute, tend to use the DLPFC in the CEN to suppress distracting information in tasks such as the Stroop task and to prevent activity in the DMN, which is associated with distraction and mind-wandering during meditation period.

In a study, the signal change in the incongruent condition for the CTRL group remained elevated until the onset of meditation, indicating significant conflict compared to other conditions.

### 4.3 Network connectivity

Functional connectivity refers to the statistical associations among anatomically distinct brain regions, including the DMN and CEN (De Filippi et al., 2022), and is commonly operationalized as temporal correlations between spontaneous or task-related fluctuations in neural activity. Utilizing CONN^3^, a dedicated toolbox for estimating functional connectivity based on temporal correlations of neural signals, an ROI-to-ROI functional network connectivity analysis was conducted among the predefined brain regions of interest. This approach provides a systematic and reproducible framework for examining interregional functional relationships. The analysis revealed a robust pattern of negative functional connectivity linking the PCC, including the precuneus (within the DMN), the PPC (within the CEN), and the lateral parietal (LP) cortices, as illustrated in Figure 6 (lower left panel; FDR-corrected threshold, *p* < 0.05), during the 48–96 s time window of the meditation condition.

Within the DMN, inhibitory connections were identified among the PCC, CEN, and lateral parietal (LP) cortex, as determined through ROI-to-ROI analysis with false discovery rate (FDR) correction, yielding a significance level of *p* < 0.05 (Figure 6, left side). Additionally, under incongruent conditions, both inhibitory and excitatory connections were observed among the lateral and medial cortices (uncorrected for multiple comparisons, *p* < 0.05: Figure 6, right side). Specifically, inhibitory connections were noted between the right PPC and ACC, as well as between the anterior insula and supramarginal gyrus, although not corrected for multiple comparisons (*p* < 0.05, not displayed here).

This study delved into the operational dynamics of the brains of long-term Zen meditating monks. The results indicate that their extensive meditation training enhances attentional control by suppressing irrelevant information, with specific regions in the prefrontal cortices playing a pivotal role. The diminished activation observed in these areas suggests optimized attentional control in seasoned monks.

### 4.4 Limitations and future directions

Several limitations of the present study should be acknowledged.

First, the sample size was relatively small, which may limit the generalizability of the findings. Future studies with larger and more diverse samples will be necessary to confirm the robustness of the present results. In addition, although overall behavioral trends were consistent with the proposed interpretation, some effects did not reach conventional levels of statistical significance, and therefore caution is warranted when interpreting their functional implications.

Second, the present behavioral and fMRI data do not fully clarify how Zen meditation differentially modulates activity within the default mode network (DMN) and the central executive network (CEN). In particular, the cross-sectional nature of the study limits strong causal inferences regarding the observed neural mechanisms. Longitudinal or training-based designs will be important for addressing this issue in future research.

Third, the use of fMRI during both conflict tasks and meditation may have constrained the temporal resolution of the observed neural processes. Future studies combining fMRI with complementary methods, such as behavioral measures or techniques with higher temporal resolution, may provide a more comprehensive understanding of the neural dynamics underlying Zen meditation (Miller, 2000; Sridharan et al., 2008; Piccolli et al., 2015; Bullmore and Sporns, 2009).

Addressing these limitations in future research will be essential for advancing our understanding of the neural correlates of Zen meditation.

## 5 Conclusion

In summary, our findings suggest how seasoned monks with long-term Zen meditation training exhibit a trend of enhanced responses and significantly reduced cognitive conflict in both the lateral and medial prefrontal cortices, as well as the ACC, compared to the control (CTRL) group. Zen meditation is generally believed to reduce DMN activity, leading to decreased MPFC and ACC activity, which in turn fosters a heightened state of awareness. However, in this study, Stroop trials showed that DMN activity was not strongly associated with increased CEN activity. Rather, the DMN and CEN share attentional resources and work together. In addition, the CEN is likely to help reduce overactivity in the DMN, especially during mind-wandering. Meanwhile, functional connectivity analysis revealed suppression in DMN-related regions during meditation in the 48–96 s time interval.

## Data Availability

The raw data supporting the conclusions of this article will be made available by the authors, without undue reservation.

## Footnotes

1
https://www.mathworks.com/help/matlab/

2
https://web.conn-toolbox.org/resources/conn-documentation/manual

3
www.nitrc.org/projects/conn
